# Fecal Microbiota Transplantation: A New Therapeutic Attempt from the Gut to the Brain

**DOI:** 10.1155/2021/6699268

**Published:** 2021-01-16

**Authors:** Hao-Ming Xu, Hong-Li Huang, You-Lian Zhou, Hai-Lan Zhao, Jing Xu, Di-Wen Shou, Yan-Di Liu, Yong-Jian Zhou, Yu-Qiang Nie

**Affiliations:** Department of Gastroenterology and Hepatology, Guangzhou Digestive Disease Center, Guangzhou First People's Hospital, School of Medicine, South China University of Technology, Guangzhou 510180, China

## Abstract

Gut dysbacteriosis is closely related to various intestinal and extraintestinal diseases. Fecal microbiota transplantation (FMT) is a biological therapy that entails transferring the gut microbiota from healthy individuals to patients in order to reconstruct the intestinal microflora in the latter. It has been proved to be an effective treatment for recurrent *Clostridium difficile* infection. Studies show that the gut microbiota plays an important role in the pathophysiology of neurological and psychiatric disorders through the microbiota-gut-brain axis. Therefore, reconstruction of the healthy gut microbiota is a promising new strategy for treating cerebral diseases. We have reviewed the latest research on the role of gut microbiota in different nervous system diseases as well as FMT in the context of its application in neurological, psychiatric, and other nervous system-related diseases (Parkinson's disease, Alzheimer's disease, multiple sclerosis, epilepsy, autism spectrum disorder, bipolar disorder, hepatic encephalopathy, neuropathic pain, etc.).

## 1. Introduction

**Figure 1:**
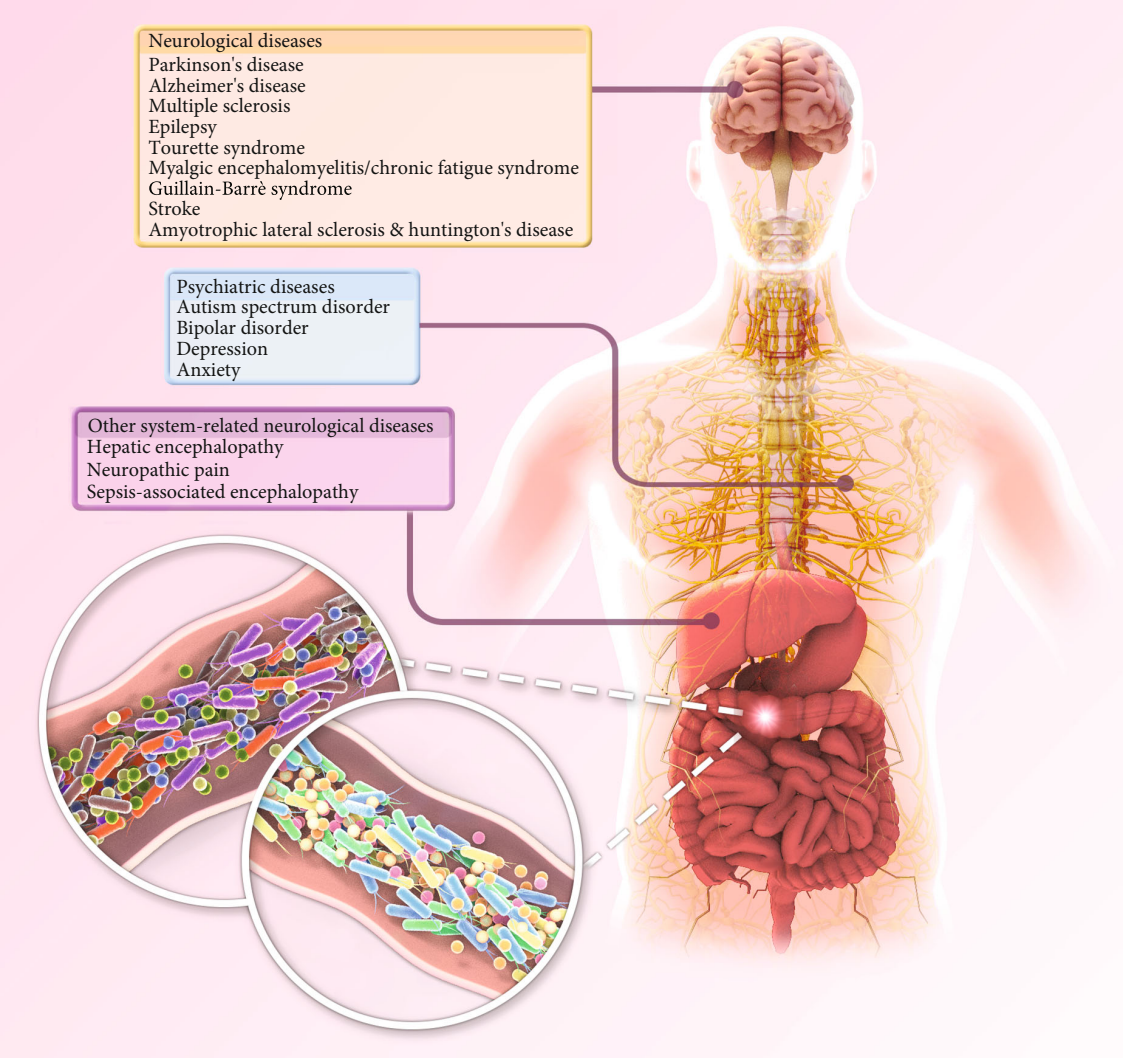
Current applications of FMT in various neurological and psychiatric diseases. Normal gut microbiota plays an important role in maintaining the functional stability of the gut-brain axis. Excessive reproduction of pathogenic bacteria or reduction of probiotics can lead to gut microbiota disorder and mediate a variety of neurological and psychological diseases. As an important therapeutic method to reconstruct gut microbiota, FMT has been tried to be applied to a variety of diseases related to gut-brain axis.

The gut microbiota is often considered an “invisible organ” that significantly affects human health and disease. More than 100 trillion microorganisms have been found in the human gastrointestinal (GI) tract, which encodes close to 3,000,0000 genes compared to the 23,000 genes within the human genome [1, 2], and are crucial for maintaining the balance between different physiological activities. The cross-talk between the GI tract and the central nervous system, commonly known as the gut-brain axis, plays an important role in the pathophysiology of neurological diseases. Studies increasingly show that dysbacteriosis can lead to or exacerbate various neurological and psychiatric disorders, such as Parkinson's disease [3, 4], Alzheimer's disease [5–7], autism spectrum disorder [8], multiple sclerosis [9], and epilepsy [10]. In addition, patients with neurological dysfunction often present GI symptoms [11], which underscores the causative role of the gut in neuropathological progression and provides a solid rationale for therapeutically targeting the gut microbiota in these diseases (Table 1). The current therapies targeting the intestinal microbiota include the use of antibiotics, probiotics, prebiotics, synbiotics, and fecal microbiota transplantation (FMT) that entails transplanting functional microbiota from healthy individuals into the GI tracts of patients. FMT can reconstruct the healthy gut microecology and improve clinical symptoms. Apart from its direct therapeutic effect in GI diseases, FMT has also been shown to improve neurological and psychological symptoms by modulating the gut-brain axis (Figure 1) [12]. In this review, we have summarized the gut microbiota in different nervous system diseases as well as the current applications of FMT in various neurological and psychiatric diseases and discussed the potential mechanisms and future directions.

## 2. Neurological Diseases

Studies [46–48] show that the GI tract and resident microbiota are susceptible to the neurological dysfunction associated with Parkinson's disease, Alzheimer's disease, multiple sclerosis, epilepsy, and stroke. The gut-brain axis is adversely affected by the destruction of intestinal epithelial barrier, loss of intestinal neurons, and overproduction of proinflammatory cytokines. In addition, gut microbial abundance and diversity undergo significant changes during neurological disorders, especially that of bacteria producing anti-inflammatory factors. FMT can significantly adjust the richness of intestinal species and restore the proportion of anti-inflammatory bacteria and is therefore increasingly being considered for treating diseases of the nervous system (Table 2).

### 2.1. Parkinson's Disease

Parkinson's disease (PD) is a progressive neurodegenerative disease characterized by accumulation of Lewy bodies [69]. PD patients often present with GI symptoms such as constipation [70]. According to the theory of intestinal origin of PD, a prion-like neurotrophic protein is misfolded into *α*-synuclein (*α*-syn) and transported from the GI tract to the central nervous system (CNS) [71]. Studies on the mouse model of PD have confirmed that *α*-syn can indeed be transferred from the gut to the brain by crossing the blood-brain barrier [72]. Consistent with this, several studies [3, 4, 73, 74] have reported considerable differences between the gut microbial composition and metabolites of healthy individuals and PD patients. Scheperjans et al. [73] compared the fecal microbiome of PD patients with that of 72 healthy controls and detected 77.6% lower prevalence of *Prevotellaceae* in the former. In addition, PD patients' postural instability and gait difficulty were positively associated with the higher abundance of *Enterobacteriaceae*, suggesting a causative association between the microbiota-gut-brain axis and progression of disease. Furthermore, Keshavarzian et al. [4] observed a greater proportion of LPS-producing proinflammatory bacteria (e.g., *Ralstonia*) and fewer bacteria producing the anti-inflammatory short-chain fatty acids (SCFAs) (e.g., *Blautia*, *Coprococcus*, *Roseburia*, and *Faecalibacterium*) in the gut of PD patients. Studies [75, 76] have shown that L-dopa can be metabolized into dopamine by gut microbial tyrosine decarboxylase, which is not easily affected by aromatic amino acid decarboxylase inhibitors such as carbidopa. In addition, the germ-free *α*-syn overexpressing (ASO) mice exhibited less severe motor and digestive symptoms (constipation), as well as lower microglia activation compared to their SPF counterparts [77], indicating that the gut microbiota is directly involved in PD's development.

Consistent with the above, Sun et al. [78] showed that FMT from healthy mice significantly improved the motor function in PD mice by mitigating intestinal inflammation and neuroinflammation and increasing the levels of dopamine and 5-hydroxytryptamine. The anti-inflammatory effects were mediated via TLR4/bk1/NF-*κ*B/TNF-*α* pathway blockade, reduced activity of microglia and astrocytes, and increased producing SCFAs. In contrast, FMT from PD mice had a pathological effect on healthy recipients. Huang et al. [49] recently reported that three rounds of FMT over a period one week improved constipation and motor symptoms such as leg tremors in a PD patient. However, the tremors recurred 2 months after FMT, whereas constipation was relieved even after 3 months.

### 2.2. Alzheimer's Disease

Alzheimer's disease (AD) is a neurodegenerative disease characterized by cognitive decline due to the loss of neurons and synapses following deposition of neurofibrillary tangles (NFT) and misfolded amyloid *β* (A*β*) protein plaques [79]. Several studies [5, 6] have shown that the gut microbiota composition in AD patients differs considerably from that of healthy elderly individuals. For instance, the AD patients have a higher relative abundance of LPS-producing bacteria such as *Burkholderiaceae*, *Staphylococcaceae*, *Porphyromonas gingivalis*, and *Propionibacterium acnes*, as well as fungi in their intestine compared to healthy controls. In addition, patients with cerebral amyloidosis (Amy+) and cognitive impairment have more proinflammatory bacteria in their feces and higher levels of circulating inflammatory cytokines (IL-6, IL-1*β*, etc.) compared to healthy individuals and Amy- patients [80–84]. Likewise, Cattaneo et al. [85] also detected higher circulating levels of IL-6, IL-1*β*, and other inflammation-related factors like CXCL2 and NLRP3, along with reduced levels of the anti-inflammatory IL-10 in Amy+ patients relative to that in controls and Amy- patients. Furthermore, the Amy+ patients showed lower abundance of *Eubacterium rectale* and a higher abundance of *Escherichia/Shigella* compared to both healthy controls and Amy- patients. A significantly positive correlation was observed between the levels of proinflammatory factors and the abundance of *Escherichia/Shigella*. Several bacterial species are known to secrete neurotransmitters and alter the expression of synaptic plasticity, which may play a role in the pathogenesis of AD [86]. In addition to these direct effects, some changes in gut microbiota may indirectly promote AD by triggering neuroinflammation [87]. Consistent with this hypothesis, there are reports that probiotics can improve cognitive function in not only animal models but also AD patients or adults with cognitive impairment [88–90]. Furthermore, the age-related decline in cognitive ability may also be related to the concomitant decrease in the number of anti-inflammatory bacteria in the human gut [91, 92].

Recent studies [93, 94] have shown that antibiotic-mediated depletion of the gut microbiota alleviated A*β*-pathology and neuroinflammation in a mouse model of AD, and the therapeutic effect of antibiotics was partially reversed following FMT from AD mice. In addition, germ-free mice receiving feces from healthy old mice had worse cognitive function compared to the recipients of feces from younger mice due to lower fecal levels of nervous system-related metabolites (such as GABA) in the former [95]. Kim et al. [96] transplanted the fecal microbiota from health control mice into the recently developed AD-like pathology with amyloid and neurofibrillary tangle (ADLPAPT) transgenic mouse model and observed a significant reduction in cerebral amyloid plaques, NFTs and reactive gliosis, which correlated to improve cognitive and memory function. Hazan [50] reported the case of an 82-year-old AD patient who showed remission of *Clostridium difficile* infection (CDI) symptoms after receiving a single FMT from his 85-year-old wife and a negative stool test 2 months later. Interestingly, the minimental state examination (MMSE) score of the patient increased from 20 (mild cognitive impairment) to 26 (normal cognitive function) 2 months after FMT, and he reported memory retention and significant improvement in mood (MMSE score 29) after 4 and 6 months, respectively.

### 2.3. Multiple Sclerosis

Multiple sclerosis (MS) is a demyelinating disease of the CNS with uncertain etiology, although genetics, infection, and environmental factors have been implicated as key pathological factors [97]. The gut microbiota regulates the production of myelin sheath in the prefrontal cortex of mice [98, 99] and maintains the integrity of the blood-brain barrier [100] by producing SCFAs [101]. This is suggestive of a dysregulated gut microbiome in MS since the loss of blood-brain barrier integrity is also a cardinal sign of this disorder. In addition to the direct role in demyelination and blood-brain barrier disruption, the gut microbiota and its metabolites also regulate neuroinflammation [102–104], although the exact relationship between gut microorganisms and MS-related neuroinflammation needs a further study. The intestinal microbiota of MS patients have a lower relative abundance of Treg cell-inducing bacteria [9, 105], which may increase the proportion of peripheral Th1 and Th17 cells [98]. In addition, the risk of relapse in MS patients is associated with the depletion of Fusobacteria, expansion of the phylum Firmicutes, and presence of Archaea (Euryarchaeota) [106]. Oral gavage with *Prevotella histicola* not only reduced the severity of symptoms in a mouse model of MS but also decreased the number of Th1 and Th17 cells, while increasing that of Treg cells [107]. A randomized control trial (RCT) on 40 MS patients showed that probiotic (*Lactobacillus acidophilus*, *Lactobacillus casei*, *Bifidobacterium bifidum*, and *Lactobacillus fermentum*) supplementation for 12 weeks significantly increased the circulating levels of IL-8 and TNF-*α* and improved the expanded disability status scale (EDSS) scores [108].

The clinical and pathophysiological characteristics of MS are best simulated in the experimental autoimmune encephalomyelitis (EAE) mouse model [109]. Oral gavage of the fecal microbiota from MS patients exacerbated the symptoms in EAE mice and decreased the levels of the anti-inflammatory cytokine IL-10 [98, 110]. Li et al. similarly showed that FMT from healthy mice alleviated the symptoms in EAE mice by reducing activity of microglia and astrocytes and restoring the blood-brain barrier integrity and axonal myelination [111]. The therapeutic effects of FMT in MS have been reported in only two studies so far [51, 52]. In one patient with secondary progressive MS complicated with recurrent CDI, FMT mitigated the recurrent infection and prevented disease progression of MS. However, the EDSS score of the patient stabilized without any improvements in the symptoms. Therefore, although FMT has limited therapeutic effect; it has the potential to provide long-term benefits for MS patients [52]. Furthermore, 3 MS patients with severe constipation were able to defecate normally after FMT, and their exercising ability was also improved significantly [51].

### 2.4. Epilepsy

Epilepsy is a chronic disease characterized by the sudden abnormal discharge from cerebral neurons, which leads to transient brain dysfunction. The individual susceptibility to epilepsy is associated with genetic and environmental factors, although the exact etiology of most cases remains unclear [16]. Nevertheless, the composition and distribution of gut microbes in patients with intractable epilepsy are distinct from that in healthy controls [17, 112, 113]. Peng et al. [17] found that compared to drug-sensitive patients, the intestinal Firmicutes/Bacteroides ratio and *α*-diversity were significantly higher in the drug-resistant patients. Interestingly, the *α*-diversity of the latter was similar to that of healthy controls, most likely due to an aberrant increase in the number of rare bacterial genera such as *Clostridium XVIII*, *Atopobium*, *Holdemania*, *Dorea*, *Saccharibacteria*, *Delftia*, *Coprobacillus*, *Paraprevotella*, *Ruminococcus*, *Gemmiger*, *Akkermansia*, *Neisseria*, *Coprococcus*, *Fusobacterium*, *Methanobrevibacter*, *Phascolarctobacterium*, and *Roseburia*. In addition, the increased abundance of *Bifidobacterium* and *Lactobacillus* was associated with fewer seizures per year, and a ketogenic diet reduced the frequency of seizures by modulating the gut microbiota [114]. Sewal et al. [115] further observed that intraperitoneal injection of LPS increased the frequency of epileptic symptoms, which was accompanied by an increase in the blood-brain barrier permeability and in the cerebral levels of proinflammatory cytokines. Antibiotics can protect against epileptic seizures by altering the bacterial population, although there is evidence that they may even induce epilepsy [16]. In addition, probiotic strains such as *Lactobacillus acidophilus*, *Lactobacillus plantarum*, *Lactobacillus casei*, *Lactobacillus helveticus*, *Lactobacillus brevis*, *Bifidobacterium lactis*, *Streptococcus salivarius subsp.*, and *Thermophilus* have also shown a positive effect in epilepsy patients [116, 117]. Olson et al. [118] observed that transplantation of ketogenic microbiota decreased the number of seizures in mice at a higher threshold. He et al. [53] reported a case of epilepsy complicated with Crohn's disease in a 17-year-old patient who showed improvements in neurological and intestinal symptoms following three rounds of FMT. Antiepileptic therapy with sodium valproate was discontinued after 20 months, and no epileptic seizures were observed.

### 2.5. Tourette Syndrome

Tourette Syndrome (TS) is a neurodevelopmental disorder characterized by motor and speech tics in childhood [119]. Liao et al. [120] found that probiotic supplementation improved tic-like behavior in mice, which coincided with an increased level of dopamine and norepinephrine. A study on 30 pediatric acute-onset neuropsychiatric syndrome and pediatric autoimmune neuropsychiatric disorders associated with streptococcal infections syndrome patients revealed a significantly different gut microbial composition compared to that of healthy controls [18]. Another study found that [121] antibiotics that effectively reduce streptococcal infections can also mitigate the associated tic disorders. Zhao et al. [54] reported that FMT eliminated involuntary articulation, reduced involuntary shrugging, and increased attention span in a pediatric case of TS over a period of 8 weeks. In an open label clinical trial [55], 11 TS patients experienced a transient decrease in seizure severity following three rounds of FMT.

### 2.6. Myalgic Encephalomyelitis/Chronic Fatigue Syndrome

Myalgic encephalomyelitis/chronic fatigue syndrome (ME/CFS) is characterized by unexplained persistent fatigue, disturbed sleep, cognitive impairment, fever, postural intolerance, lymphadenopathy, and irritable bowel syndrome. The gut microbiota is significantly altered in patients with ME/CFS [19], and the extent of microbial dysbiosis affects disease severity [122]. Sheedy et al. [123] observed increased relative abundance of gram-positive lactic acid-producing bacteria in the gut of ME/CFS patients, which may lower the mucosal pH and increase permeability. Moreover, the transfer of lactic acid from intestine to the blood may be one of the reasons for the increase of lactate level in cerebrospinal fluid of ME/CFS patients [124–126]. Selective transplantation of 13 nonpathogenic enteric bacteria through colonoscopy [56] significantly improved intestinal and other symptoms in 42/60 ME/CFS patients. In addition, 7/12 patients who were followed up for 15 to 20 years showed complete remission, indicating FMT is a promising treatment for ME/CFS.

### 2.7. Guillain-Barré Syndrome

Guillain-Barré Syndrome (GBS) is a paralytic autoimmune neuropathy caused by infection, especially *Campylobacter jejuni* infection in the GI tract, or other immune stimulation [127]. The innate immune response to campylobacteriosis is characterized by the accumulation of neutrophils and macrophages, inflammatory damage to the mucosa, gut barrier defects, and malabsorption, which eventually lead to bloody diarrhea [23]. Mice inoculated with *Campylobacter jejuni* from GBS patients showed increased levels of autoantibodies and peripheral nerve injury [128, 129], indicating a close association between gut dysbiosis and GBS pathogenesis. In fact, the cross-reaction between LPS produced by *Campylobacter jejuni*, and the peripheral gangliosides is one of the causative factors of GBS [130]. The combination of antibiotics and FMT significantly expedited *Campylobacter jejuni* clearance from the infected mice [131]. In addition, Brooks et al. [132] observed that human FMT increased the Th2 and autoimmune response in mice infected with *Campylobacter jejuni*. Finally, the outer core LPS of *Campylobacter jejuni* can directly initiate the peripheral neuropathy of GBS by inducing production of neurotoxic antiganglioside autoantibodies [133].

### 2.8. Stroke

Stroke is an acute cerebrovascular accident characterized by muscular and sensory weakness. Studies show that the composition of gut microbiota of stroke patients differs considerably from that in healthy controls [134, 135], although there are some reports indicating transient or no change [136]. Furthermore, the possible role of gut dysbiosis in stroke is ambiguous [137]. One study showed that a stroke episode decreased intestinal motility and *α*-diversity and led to bacterial overgrowth, intestinal barrier damage, and increased infiltration of inflammatory immune cells in the gut-associated lymphoid tissue and brain, eventually increasing the infarct volume [138]. In addition, the translocation of gut microbiota and their metabolites may also be involved in the pathogenesis of stroke [139]. For instance, trimethylamine-N-oxide produced by gut microbiota may be associated with a higher risk of atherosclerosis-mediated cardiovascular events, including stroke [140, 141]. Prebiotic treatment exacerbated the functional damage and inflammation in a mouse model of stroke, which increased the infarct volume [138, 142]. However, transplantation of healthy microbiota reduced infarct volume [138], indicating that FMT can be considered for treating stroke patients.

### 2.9. Amyotrophic Lateral Sclerosis and Huntington's Disease

Amyotrophic lateral sclerosis (ALS), also known as motor neuron disease (MND), is a neurodegenerative disorder characterized by progressive atrophy of the limb, trunk, chest, and abdomen muscles following upper and lower motor neuron injury [143]. The mouse model of ALS shows an altered gut microbiota structure compared to healthy mice, such as a lower relative abundance of butyrate-producing bacteria [144]. Although a definitive pathological role of the gut microbiota in ALS has not been reported in humans [26], the clinical potential of FMT is still being explored [145]. Huntington's disease is caused by an autosomal dominant mutation in the huntingtin gene and is inherited in most cases. Nevertheless, several studies have implicated nongenetic factors in the development of Huntington's disease, such as the gut microbiota. Metabonomics analysis of the sera of preonset and early onset Huntington's disease patients and healthy controls showed significant differences in the gut microbiota metabolites across all groups [146], indicating that changes in the microflora determine disease course. The role of gut dysbiosis in the pathogenesis of Huntington's disease has also been confirmed in a murine transgenic model [29]. However, further studies are needed to fully understand the causative role of the gut microbiota and its metabolites in the genesis, progression, and severity of Huntington's disease.

## 3. Psychiatric Diseases

There is growing evidence that gut dysbiosis also contributes to mental health and psychiatric disorders, such as autism spectrum disorder, bipolar disorder, depression, anxiety, obsessive-compulsive disorder, posttraumatic stress disorder, schizophrenia, and dementia through the gut-brain axis [147, 148]. FMT has gained attention as a viable therapeutic option for these conditions (Table 2).

### 3.1. Autism Spectrum Disorder

Autism spectrum disorder (ASD) is a group of neurodevelopmental disorders characterized by changes in social interaction and repetitive, stereotypical behavior [149]. Recent studies show that gut microbial community and metabolites of ASD patients are distinct from that of healthy individuals [8, 150]. Although a putative relationship between gut dysbiosis and ASD behavior has been established in rodent models and human subjects, studies have not been sufficient to confirm the causal relationship between gut microbiota and ASD symptoms. The predominant phyla of the healthy adult human gut are Bacteroidetes (e.g., *Bacteroides* and *Prevotella*), Firmicutes (e.g., *Clostridium*, *Lactobacillus*, and *Ruminococcus*), Proteobacteria (e.g., *Enterobacter*), and Actinobacteria (e.g., *Bifidobacterium*) and constitute more than 90% of the gut microbiota [151, 152]. Since germ-free mice are socially dysfunctional compared to wild-type mice, the gut microbiota likely play an important role in normal behavior [153]. Regular administration of probiotics including *Lactobacillus reuteri*, *Lactobacillus plantarum*, *Lactobacillus acidophilus*, *Lactobacillus rhamnosus*, and *Bifidobacterium longum* over a period of 3 weeks to 6 months improved autistic symptoms significantly in ASD children [154–156]. *Lactobacillus rhamnosus* and a placebo were, respectively, administered to 40 and 35 infants for the first 6 months of life in an RCT, and all subjects were followed over 13 years. Six infants of the placebo group were diagnosed with Asperger's syndrome or attention deficit hyperactivity disorder during the follow-up period whereas none in the probiotic group exhibited any signs of autism, indicating that early administration of probiotics can potentially reduce the risk of developing ASD [155]. A recent study has shown that there is a causal relationship between maternal diet, changes in gut microbiota, and social behavior. Among female neonatal rats fed with a high-fat diet, female rats born on a high-fat diet and for more than 4 weeks *Lactobacillus reuteri* restored gut microbial diversity and significantly improved their social behavior [157].

Sharon et al. [158] found that germ-free mice transplanted with the feces from children with ASD exhibited similar symptoms. In addition, the offspring of these FMT recipients also experienced these symptoms and showed alternative splicing of ASD-related genes in the brain. Likewise, FMT from healthy hamsters alleviated the ASD-like symptoms in the autism hamster model [159] by alleviating the brain oxidative stress response. In an open clinical trial on 18 children with ASD, FMT for 7 to 8 weeks could significantly improve digestive symptoms (abdominal pain, constipation, diarrhea, and indigestion) and the behavioral symptoms [32]. Furthermore, FMT also improved the bacterial diversity by significantly increasing the abundance of *Bifidobacterium*, *Desulfovibrio*, and *Prevotella*. The therapeutic effects persisted for 8 weeks after ceasing treatment. In another study, the ASD symptoms improved in 8/9 recipients of FMT and antibiotic treatment [57]. Zhao et al. [58] conducted an open label RCT on 24 autistic and 24 normal children that were treated with FMT for 2 months. Although FMT improved the behavioral and GI symptoms, the effects were transient.

### 3.2. Bipolar Disorder

Bipolar disorder (BD) is a type of mood disorder that clinical manifests as distinct episodes of depression, manic seizures, and their combination. Both the diversity and taxonomic composition of the gut microbiota in BD patients are significantly different from that of healthy individuals [36]. Painold et al. [160] further showed that the phylum Actinobacteria and class *Coriobacteria* were significantly more abundant in the gut of BD patients, whereas *Ruminococcaceae* and *Faecalibacterium* were more abundant in the healthy controls as per 16S rRNA gene sequencing and LEfSE analysis. They also observed a negative correlation between microbial *α*-diversity and duration of BD and identified bacterial clades associated with inflammatory status, serum lipids, depressive symptoms, oxidative stress, anthropometrics, and metabolic syndrome in the BD patients. Hu et al. [35] analyzed the gut microflora of 52 BD patients and 45 controls and found that the *α*-diversity of untreated BD patients was lower than that of the control group, and the predominant phyla were Bacteroidetes and Firmicutes, respectively. In addition, butyrate-producing bacteria were less abundant in the untreated patients, which was restored following quetiapine treatment. Furthermore, probiotics supplementation for a period of 3 months improved the cognitive and executive functions of 20 BD patients [161].

Hinton [59] reported a case of a 29-year-old female patient diagnosed with type I DSM-IV BD who had been treated with various drugs, including lithium, lamotrigine, valproate, quetiapine, olanzapine, and various benzodiazepines, that led to significant weight gain and poor quality of life. Nine rounds of FMT in 11 months not only alleviated depression and mania but also helped her lose the excess weight and remain asymptomatic without using other drugs.

### 3.3. Depression

Depression is a common mental disease typically characterized by persistent feelings of sadness and loss of interest in daily activities. It results from a combination of both genetic and environmental factors, and a major cause is stress [162]. Studies increasingly show that the gut microbiota can shape cognition through the microbiota gut-brain axis, and mice with altered microbiota usually exhibit depression-related behaviors [163]. Kelly and Borre [164] analyzed the intestinal flora of 34 patients with depression and 33 matched healthy subjects and found that the microbial abundance and biodiversity were decreased in the patient group. FMT from these patients into germ-free rats induced depression-like behavior such as lack of pleasure and anxiety in the latter, along with increased levels of tryptophan. A meta-analysis of 71 studies published between 2003 and 2019 [165] further revealed that probiotics and prebiotics can significantly improve symptoms of anxiety and depression compared to untreated or placebo-treated controls and provide additional benefits to patients with other diseases such as irritable bowel syndrome.

Zhang et al. [163] found that FMT from depressed patients into germ-free mice led to depressive behavior in the latter. Similar results were observed after antibiotic treatment as well. Furthermore, FMT from the NLRP3-knockout mice significantly improved the behavioral symptoms in a mouse model of depression. Likewise, Xie [166] also found that the fecal microbiota of healthy mice alleviated depressive symptoms. In a recent case report [60] of an older woman diagnosed with depression, a single FMT improved sleep cycle, appetite, and general mood within 4 days of treatment. The patient was able to live independently after 2 weeks and showed an increase in weight. Six months later, her weight had returned to normal, constipation symptoms had improved, and the Patient Health Questionnaire-9 score decreased from 21 to 4.

### 3.4. Anxiety

Anxiety is one of the most common types of neurosis and is characterized by feelings of tension/worry without a clear objective, restlessness, and autonomic nerve dysfunction. Clinically, it is classified into chronic/generalized anxiety and acute anxiety or panic attack [167]. A large case-control study [168] showed that the use of antibiotics increased the risk of anxiety and depression, and the risk increased with the frequency of usage, suggesting a causative or ancillary role of the gut microbiota. Furthermore, there is evidence that depression can lead to secondary changes in the composition of the gut microbiota, resulting in a regulatory feedback loop between depression and dysbacteriosis [169]. Compared to the SPF mice, sterile mice showed significantly higher anxiety in the elevated maze test, and oral administration of the JB-1 probiotic strain effectively reduced the anxious behavior and improved performance. Furthermore, a systematic review of 21 studies including 1503 subjects with anxiety disorders concluded that microbiota-targeted therapies [41], including probiotics supplements, single probiotics, double probiotics, multiple probiotics, dietary fiber supplement, and low FODMAP diet, can alleviate symptoms of anxiety by regulating the gut microbiota.

De Palma et al. [170] transplanted fecal microbiota from healthy control and diarrhea-predominant irritable bowel syndrome (IBS) patients with (IBS-A) or without anxiety into germ-free mice and analyzed the changes in intestinal function and behavior. The gut microbiota of mice transplanted with the feces of IBS patients showed unique clustering characteristics compared to that of control fecal recipients. Anxiety-like behavior was determined with the light/dark preference test and platform jumping test, which showed that the IBS-A recipient mice had the least preference for light and showed the delay in jumping off a high platform, both of which are indicative of a higher degree of anxiety. These studies clearly indicate the involvement of gut dysbiosis in the severity of anxiety symptoms.

## 4. Other System-Related Neurological Diseases

Several neurological and psychological diseases are frequently complicated with digestive system symptoms. Likewise, some diseases predominantly affecting the nonnervous systems may also have a neurological component and are commonly manifested as encephalopathies. For instance, decompensated hepatic encephalopathy and peripheral neuropathy are severe complications of cirrhosis and diabetes, respectively, and sepsis patients often present delirium, coma, and other neurological symptoms. The role of the gut microbiota in these encephalopathies is increasingly being recognized, thereby indicating the therapeutic potential of FMT for these diseases (Table 2).

### 4.1. Hepatic Encephalopathy

Hepatic encephalopathy (HE) is a serious complication of cirrhosis and is caused by brain dysfunction. The increased content of hepatic ammonia in cirrhosis patients with mild HE indicates the pathological involvement of intestinal dysbiosis. For instance, the intestinal tract of cirrhotic patients with/without mild HE frequently harbors urease-positive *Streptococcus salivarius*, which is absent in healthy individuals [171]. Thus, *S. salivarius* is a promising therapeutic target in liver cirrhosis patients with mild HE. Sung et al. [43] confirmed that fecal microbiota can predict the clinical prognosis of patients with liver cirrhosis and HE, such as *Lactobacillus*, *Bacteroides*, *Clostridium_incertae_sedisof*, and *Clostridium XI*, which were associated with patients' mortality. Furthermore, Kawaguchi et al. [172] showed that rifaximin improved both liver and neuropsychological function in liver cirrhosis patients with HE by adjusting the gut microbial structure.

A promising case study of a 57-year-old patient with HE due to alcoholic and hepatitis C cirrhosis [61] showed that FMT in addition to lactulose objectively improved reaction time, serum ammonia, and quality of life scores. However, these improvements were transient and subsided to the baseline levels within 7 weeks of FMT cessation. Furthermore, Bajaj et al. [62] conducted an RCT on male cirrhotic patients diagnosed with recurrent HE and found that FMT reduced hospitalization rate and improved cognitive ability in these patients during the 5-month follow-up. In another clinical trial conducted by Bajaj et al. [63], administration of FMT capsules to HE patients restored the gut microflora by significantly increasing the abundance of *Bifidobacterium* and *Ruminococcaceae* and decreasing that of pathogenic genera like *Streptococcus* and *Veillonella*. The FMT-induced changes in the gut microbiota led to an increase in duodenal E-cadherin and defensin-*α*5 expression and reduced serum levels of IL-6 and LBP.

### 4.2. Neuropathic Pain

Neuropathic pain is caused by peripheral or CNS injury (such as nerve injury or chemotherapy injury) or diabetes and is characterized by abnormal sensations or pain even after normal stimulations [173]. The composition and function of the gut microbiota in diabetic patients differ significantly from that of healthy controls [174]. FMT from conventionally reared mice increased the insulin resistance in germ-free mice [175], whereas subjects with metabolic syndrome showed increased insulin sensitivity following FMT [176]. Gut microbiota can also directly regulate the excitability of spinal dorsal root neurons or indirectly regulate inflammation in the peripheral and central nervous system [177]. Oxaliplatin can cause peripheral neuropathy and pain, but this phenomenon is not obvious in mice with antibiotic cleaning or in mice with complete loss of gut microbiota. Furthermore, if FMT was performed on the appellate mice, the pain would be restored, indicating that the gut microbiota has an effect on neuropathic pain [178]. Another study found that probiotics alleviated the characteristics of paclitaxel-induced neuropathic pain *in vitro* [179], although their efficacy is dependent on the type of neuropathic pain. For instance, *Lactobacillus Reuteri* or *Bifidobacterium* were not effective against the neuropathic pain induced by chronic compression injury in rats [180].

A case study [64] of a woman with type 2 diabetes mellitus and diabetic neuropathy showed that two rounds of FMT improved limb pain and paresthesia, which was manifested as decreased visual analogue pain score (VAS) and increased tibial nerve motor conduction velocity, without any significant improvement in EMG sensory dysfunction. In addition, the fasting blood glucose level also decreased and stabilized, and glycosylated hemoglobin content decreased post-FMT.

### 4.3. Sepsis-Associated Encephalopathy

Sepsis is an acute systemic infection caused by various pathogenic bacteria that invade the bloodstream and rapidly proliferate and produce life-threatening toxins. Sepsis-associated encephalopathy is a key neurological manifestation of sepsis, with symptoms ranging from delirium to coma. It occurs in almost 70% of the ICU patients and is associated with higher ICU and hospital mortality, as well as poor long-term outcomes (including cognitive and functional outcomes) [181, 182]. The toxins and other harmful antigens secreted by pathogenic bacteria or viruses can be neutralized by the antibodies produced by antigen-primed B cells. Intestinal microorganisms have been shown to induce the clonal expansion of specific B cell populations and increase production of antibodies to prevent the spread of infection [183]. Li et al. found that FMT effectively improved the spatial memory and EEG abnormalities in an LPS-induced rat model of sepsis combined with cervical vagotomy, and the therapeutic effect of FMT was likely mediated through the vagus nerve [184]. In addition, several case reports indicate that non-CDI sepsis patients with prolonged ICU stay and complications including bacteremia, MDR bacterial infection, respiratory failure, and organ dysfunction significantly benefitted from FMT. A total of 5 patients received FMT, of which 4 showed clinical improvement and 1 died from non-FMT-related causes [65–68].

## 5. Discussion

Nervous system diseases are highly complex and show cognitive, motor, and even systemic manifestations. Given that gut dysbiosis is a potential causative factor of neurological dysfunction, FMT-mediated restoration of the gut microbiota can stall the symptoms or progression of nervous system diseases through immune, endocrine, metabolic, and/or neural pathways. The metabolites and cytokines produced by gut bacteria determine intestinal and systemic inflammation and, therefore, the intestinal barrier function. However, there are several limitations of using FMT in treating neurological, mental, and psychological diseases: (1) for many diseases, the therapeutic effects of FMT are limited to animal models and isolated cases. Although transplantation of human feces to animal models has shown encouraging results, the GI and physiological differences between humans and animals preclude the extrapolation of the results to sick or healthy humans. (2) The fecal feeding behavior often observed in mice [185] may also affect the microbiota analysis and the efficacy of FMT. In addition, animals housed in the same cage may have a closer gut microbial structure, which can also affect the results. (3) The efficacy of FMT depends on the types of antibiotics, microbial composition, intervention procedure, and donors. The exact influence of these factors and the potential adverse effects of FMT are currently unknown due to lack of long-term follow-up and appropriate controls. Therefore, it is crucial to establish scientific standards in order to gauge the therapeutic efficacy of FMT [186]. (4) The role of the gut microbiota in the early development of nervous system also needs to be elucidated. For instance, a study on 39 infants showed that the *α*-diversity of gut microbiota was also associated with functional connectivity between the auxiliary motor area and the inferior parietal lobule, and this functional connectivity affects the cognitive level at 2 years of age [187]. (5) Many successful cases of FMT in the treatment of neurological diseases/psychiatric diseases often have obvious GI symptoms, and the improvement of neurological symptoms/mental symptoms is also related to the GI symptoms. For patients with neurological diseases/psychiatric diseases but without obvious GI symptoms, whether the curative effect of FMT will be reduced or unchanged is also worth our concern.

Despite the promising results, the rationale for the clinical application of FMT is currently based on animal models and a few case reports and clinical studies. Large-scale randomized double-blind controlled trials are still needed to clarify the role of FMT in neurological diseases. At present, 33 clinical trials are ongoing on the potential therapeutic effects of FMT on mental and nervous system diseases (Table 3). Furthermore, the modes of delivering fecal microbiota also need to be improved. While a capsular form is more comfortable for the patients, fecal bacterial liquid in the form of washed/selective microbiota transplantation [188, 189] may be more effective in reducing the potential side effects.

## Figures and Tables

**Table 1:** Characteristics, consequences, and application level of FMT in neuropsychological diseases.

Disease types	Alterations of gut microbiota	Altered substances caused by microbial dysbiosis	Application level of FMT	References
*Neurological diseases*				
Parkinson's disease	Increase in *Verrucomicrobiaceae*, *Ruminococcaceae*, *Proteobacteria*, *Clostridiaceae*, *Enterobacteriaceae*, *Bifidobacteriaceae*, *Lactobacillaceae*, *Pasteurellaceae*, *Christensenellaceae*, *Lactobacilli*, *Akkermansia*, *Ralstonia* Decrease in *Firmicutes*, *Prevotellaceae*, *Coprococcus*, *Bacteroides fragilis*, *Blauti*, *Roseburia*, *Faecalibacterium*	*α*-Synuclein, LPS, SCFAs, hydrogen production	Patient & animal	[4, 13, 14]
Alzheimer's disease	Increase in *Escherichia*, *Shigella*, *Chlamydia pneumoniae*, *Borrelia burgdorferi*, *Treponema pallidum*, *Burkholderiaceae*, *Staphylococcaceae*, *Porphyromonas gingivalis*, *Propionibacterium acnes* Decrease in *Eubacterium rectale*, *Bacteroides fragilis*	Inflammatory cytokines (IL-6, CXCL2, NLRP3, IL-1*β*, IL-10), A*β*, GABA, BDNF, DHA	Patient & animal	[5, 6, 13, 15]
Multiple sclerosis	Increase in *Firmicutes*, *Clostridium*, *Escherichia Shigella* Decrease in *Bacteroides*, *Faecalibacterium*, Eubacterium rectale, *Corynebacterium*, *Fusobacteria*	Proinflammatory cytokines, butyrate, lipid 654	Patient & animal	[13]
Epilepsy	Increase in *Firmicutes*, *Proteobacteria*, *Clostridium*, *Cronobacter*, *Akkermansia*, *Ruminococcus*, *Coprobacillus*, *Clostridium XVIII*, *Atopobium*, *Holdemania*, *Dorea*, *Saccharibacteria*, *Delftia*, *Paraprevotella*, *Gemmiger*, *Neisseria*, *Coprococcus*, *Fusobacterium*, *Methanobrevibacter*, *Phascolarctobacterium*, *Roseburia* Decrease in *Bacteroidetes*, *Actinobacteria*, *Prevotella*, *Bifidobacterium*	Proinflammatory cytokines (TNF*α*, IL-6, IL-1*β*), dopamine receptors D1 and D2	Patient & animal	[16, 17]
Tourette Syndrome	Increase in *Bacteroidetes*; in particular, *Bacteroides*, *Odoribacter*, and *Oscillospira* were identified as potential microbial biomarkers	SCFAs, D-alanine, tyrosine, dopamine	Patient & animal	[18]
Myalgic encephalomyelitis/chronic fatigue syndrome	Increase in *Roseburia*, *Holdemania*, *Enterococcus*, *Streptococcus spp.* Decrease in most *Bacteroidetes* genera.	Lactic acid, LPS, LPS-binding protein, soluble CD14, oxidative stress	Patient & animal	[19–21]
Guillain-Barré Syndrome	*Campylobacter jejuni* infection is associated with GBS while *Enterococcus faecalis* as a potential protective role	LPS, peripheral nerve gangliosides	Animal	[22, 23]
Stroke	Decreased neuronal injury and improved cognitive performance were observed in diabetic mice with bilateral common carotid arteries occlusion after receiving *Clostridium butyricum*	Trimethylamine N-oxide	Animal	[24, 25]
Amyotrophic lateral sclerosis	Increase in *Dorea* Decrease in *Butyrivibrio fibrisolvens*, *Firmicutes*, *Peptostreptococcus*, *Escherichia coli*, *Oscillibacter*, *Anaerostipes*, *Lachnospira*	Butyrate	Animal	[26–28]
Huntington's disease	Increase in *Bacteroidetes* Decrease in *Firmicutes*, *Lachnospiraceae*, *Akkermansiaceae*	Methionine, glycine	Animal	[29, 30]
*Psychiatric diseases*				
Autism spectrum disorder	Increase in *Bacteroides*, *Barnesiella*, *Clostridium*, *Roseburia* Decrease in *Bifidobacterium*, *Coprococcus*, *Dialister*, *Faecalibacterium*, *Prevotella*, *Streptococcus*	Butyrate, lactate	Patient & animal	[31–33]
Bipolar disorder	Increase in *Bacteroidetes*, *Actinobacteria*, *Coriobacteria*, *Lachnospira*, *Enterobacteriaceae*, *Flavonifractor* Decrease in *Firmicutes*, *Ruminococcaceae*, *Roseburia*, *Faecalibacterium*, *Coprococcus*	Butyrate	Patient & animal	[34–37]
Depression	Increase in *Enterobacteriaceae*, *Prevotella*, *Klebsiella*, *Alistipes* Decrease in *Lachnospiraceae*, *Faecalibacterium*, *Coprococcus*, *Dialister*, *Ruminococcus*, *Lactobacillus*, *Bifidobacterium*	Butyrate, inflammatory cytokines	Patient & animal	[36, 38, 39]
Anxiety	Increase in *Fusobacterium*, *Ruminococcus*, *Escherichia Shigella* Decrease in *Faecalibacterium*, *Eubacterium*, *Sutterella*		Animal	[40, 41]
*Other system-related neurological diseases*				
Hepatic encephalopathy	Increases in *Enterobacteriaceae*, *Streptococcaceae*, *Porphyromonadaceae*, *Staphylococcaceae*, *Enterococcaceae* Decrease in *Lachnospiraceae*, *Ruminococcaceae*, *Rikenellaceae*, *Clostridium XIV*, *Phascolarctobacterium*	Ammonia, urease, SCFAs, aromatic amino acids	Patient & Animal	[42, 43]
Neuropathic pain	Associated: *Lactobacillus fermentum KBL374* & *KBL375*, *Bacteroides fragilis*, *Escherichia coli*, *Lactobacillus*, *Streptococcus* spp., *Enterococcus* spp., *Corynebacterium glutamicum*, *Peptostreptococcus*, *Clostridium sporogenes*	LPS, bacterial flagellin, indole, SCFAs, PUFAs, BAs	Patient & animal	[44]
Sepsis-associated encephalopathy	Associated: absence of anaerobes, including *Staphylococcus species* and *Escherichia coli*, with CDI, high relative abundance of pathogenic gram negatives, and *Enterococci*	LPS, SCFAs, BAs	Patient & animal	[45]

LPS: lipopolysaccharide; SCFAs: short-chain fatty acids; IL-6: interleukin-6; CXCL2: C-X-C motif chemokine ligand 2; NLRP3: recombinant NLR family, pyrin domain containing protein 3; IL-1*β*: interleukin-1*β*; IL-10: interleukin-10; A*β*: amyloid *β*-protein; GABA: *γ*-aminobutyric acid; BDNF: brain-derived neurotrophic factor; DHA: docosahexaenoic acid; TNF*α*: tumor necrosis factor-*α*; PUFAs: polyunsaturated fatty acid; Bas: bile acids.

**Table 2:** Clinical application for FMT based on the gut-brain axis.

Disease type	Studies	Study type	*N*	Location	Age	Sex	Complication	Administration route	FMT frequency	Donor	Clinical outcome
*Neurological diseases*											
Parkinson's disease	Huang et al. [49]	Case report	1	China	71	M	PD, constipation	TET tube (colon)	3 times	Healthy volunteer	Constipation cured, PD symptoms relieved for 2 months
Alzheimer's disease	Hazan [50]	Case report	1	USA	82	M	AD, rCDI	Colonoscopy	Once	His wife	MMSE score increased from 20 to 29
Multiple sclerosis	Borody et al. [51]	Case series	3	Australia	30/29/80	M/M/F	MS, constipation, vertigo, impaired concentration/MS, constipation/MS, constipation, proctalgia fugax, difficulty in walking	NA	5 FMTs/10 FMTs/5 FMTs	NA	Constipation resoluted, MS improved, 15 years post-FMT without relapse/constipation resolved, neurological symptoms improved, 3 years maintained normal motor/bowel symptoms resoluted, neurological improved
	Makkawi et al. [52]	Case report	1	Canada	61	F	MS, rCDI	Enema	Once	Her partner	rCDI resolved, prevented MS progression for over 10 years
Epilepsy	He et al. [53]	Case report	1	China	22	F	Epilepsy, CD	TET tube (colon)	3 times	Healthy volunteer	Seizure-free without antiepileptic drugs, decreasing CDAI to 104 points after 12 months and maintained until the end of 20-month follow-up
Tourette Syndrome	Zhao et al. [54]	Case report	1	China	9	M	TS	Gastroscope & colonoscopy	Once	Healthy volunteer	YGTSS-total tic score decreased from 31 to 5, motor severity score fell from 16 to 5, vocal severity score fell from 15 to 0, shifting from severe to mild
	Ding et al. [55]	Open-label clinical trial	11	China	19.2 ± 7.4	M	TS	TET tube (nasojejunal)	3 times	Healthy volunteer	45.5% (5/11), 45.5% (5/11) and 36.4% (4/11) of patients achieved improvement (≥30% reduction in YGTSS-total tic score) at week 1, week 4, and week 8 post-FMT, respectively. GTS-QoL score decreased at week 8 post-FMT
Myalgic encephalomyelitis/chronic fatigue syndrome	Borody et al. [56]	Larger cohort study	60	Australia	55.0 ± 11.5	36 F, 24 M	CFS (52 with IBS, 4 with constipation)	Single TC infusion (*n* = 5), two-day infusion (TC and enema, *n* = 52), three-day infusion (TC, 2-day enema, *n* = 3)	Once/twice/3 times	13 nonpathogenic enteric bacteria from healthy individual	35/60 patients responded after single FMT while7 patients responded after secondary FMT, giving a total of 42/60 improved patients
*Psychiatric diseases*											
Autism spectrum disorder	Ward et al. [57]	Case series	9	Canada	7.7 ± 5.4	NA	ASD	Capsules & enema	Twice	Healthy volunteer	ASD symptoms were not changed in the 21-year-old subject, while markedly improving in 1 of two 8-year-old subjects
	Zhao et al. [58]	Open-label, randomized waitlist-controlled trial	48	China	NA	NA	ASD	Gastroscope & colonoscopy	Twice	Healthy volunteer	CARS score in the FMT group showed a statistically 10.8% decrease compared to a 0.8% decrease in the waitlist group after the first FMT and remained marginally reduced after the second FMT
	Kang et al. [32]	Open-label clinical trial	18	USA	7 to 16 years	NA	ASD	Oral vs. enema	For 7–8 weeks	Healthy volunteer	80% reduction of GI symptoms post-FMT, ASD symptoms improved significantly and remained improved 8 weeks post-FMT
	Kang et al. [32]	Follow-up of a clinical trial	18	USA	7 to 16 years	NA	ASD	Oral vs. enema	For 7–8 weeks	Healthy volunteer	Two years post-FMT, most GI symptom improvements continued, and autism-related symptoms improved even more
Bipolar disorder	Hinton [59]	Case report	1	Australia	33	F	BD	NA	9 FMTs over a period of 2 months	Her husband	Symptom-free from depression
Depression	Cai et al. [60]	Case report	1	China	79	F	MDD	Gastroscope	Once	Her grandson	PHQ-9 scores improved
*Other system-related neurological diseases*											
Hepatic encephalopathy	Kao et al. [61]	Case report	1	Canada	57	M	Liver cirrhosis, HE	Colonoscopy & enema	5 FMTs	Healthy volunteer	Stoop test, serum ammonia, and quality of life all significantly improved; appetite, alertness and overall well-being improved
	Bajaj et al. [62]	Open-label, randomized clinical trial	20	USA	64.5 ± 5.1 (FMT) vs. 62.9 ± 9.8 (SOC)	M	Liver cirrhosis, HE	Enema	Once	Healthy volunteer	Significantly improved in PHES total score and EncephalApp Stroop in the FMT group
	Bajaj et al. [63]	A phase 1, randomized, placebo-controlled trial	20	USA	63.3 ± 4.2 (FMT) vs. 64.2 ± 6.2 (SOC)	16 M, 4 F	Liver cirrhosis, HE	Capsules	15 capsules of FMT/placebo	Healthy volunteer	EncephalApp improved
Neuropathic pain	Cai et al. [64]	Case report	1	China	46	F	Diabetic neuropathy	Colonoscopy	Twice	Healthy volunteer	The glycemic control improved, with a remarkable relief of the symptoms of painful DN
Sepsis	Li et al. [65]	Case report	1	China	29	F	Bacteremia, shock	Nasoduodenal tube	Once	Healthy volunteer	Fever went down, and the stool output had a marked reduction
	Li et al. [66]	Case report	1	China	44	F	Shock, respiratory failure, AKI	Nasoduodenal tube	Once	Healthy volunteer	Patient's septic symptoms and severe diarrhea were successfully controlled
	Wei et al. [67]	Case report	2	China	65/84	M	Shock, respiratory failure, bacteremia, AKI	Nasoduodenal tube	Once	Healthy volunteer	MODS and severe diarrhea were alleviated in both patients
	Gopalsamy et al. [68]	Case report	1	Georgia	57	M	MDRO infection, respiratory failure	PEG tube	Once	NA	Death (not due to FMT)

FMT: fecal microbiota transplantation; M: male; F: female; PD: Parkinson's disease; TET: transendoscopic enteral tubing; MMSE score: minimental state examination score; AD: Alzheimer's disease; rCDI: recurrent clostridium difficile infection; CDAI: Crohn's disease activity index; MS: multiple sclerosis; CD: Crohn's disease; YGTSS-total tic score: Yale Global Tic Severity Scale-total tic score; TS: Tourette Syndrome; GTS-QoL score: Gilles de la Tourette Syndrome-quality of life score; CFS: chronic fatigue syndrome; IBS: Irritable bowel syndrome; TC: transcolonoscopic; ASD: autism spectrum disorder; CARS score: childhood autism rating scale score; BD: bipolar disorder; MDD: major depressive disorder; PHQ-9 scores: Patient Health Questionnaire-9 scores; HE: hepatic encephalopathy; PHES total score: psychometric hepatic encephalopathy score total score; DN: diabetic neuropathy; AKI: acute kidney injury; MODS: multiple organ dysfunction syndrome; MDRO infection: multidrug-resistant organism infection; PEG tube: polyethylene glycol tube.

**Table 3:** Clinical trials of FMT involving in nervous and mental disease.

NCT number	Conditions	FMT route	Phases	Status	Locations
NCT02255617	Hepatic encephalopathy	Colonoscopy & enema	Phase 1, phase 2	Completed	Canada
NCT02636647	Hepatic encephalopathy	Enema	Phase 1	Completed	United States
NCT03420482	Hepatic encephalopathy	Capsules	Phase 2	Recruiting	United States
NCT03152188	Hepatic encephalopathy	Capsules	Phase 1	Completed	United States
NCT03439982	Hepatic encephalopathy	Colonoscopy & enema	Phase 1, phase 2	Recruiting	Canada
NCT03796598	Hepatic encephalopathy	Capsules & enema	Phase 1, phase 2	Recruiting	United States
NCT03408886	Autism spectrum disorder	Pill (no detail)	Phase 2	Recruiting	United States
NCT03426826	Autism spectrum disorder	Gastroscope	Phase 1	Recruiting	United States
NCT03829878	Autism spectrum disorder	Capsules	Phase 2	Not yet recruiting	United States
NCT04182633	Autism spectrum disorder	Oral administration of FM (no detail)	Phase 2	Recruiting	United States
NCT04246398	Children with autism	Capsules	Not applicable	Not yet recruiting	Israel
NCT03026231	Parkinson's disease	Capsules	Phase 1, phase 2	Withdrawn	United States
NCT03671785	Parkinson disease	Capsules	Phase 1	Recruiting	United States
NCT03808389	Parkinson disease	Nasojejunal	Not applicable	Recruiting	Belgium
NCT03876327	Parkinson disease	Not applicable	Phase 2, phase 3	Completed	Israel
NCT03183869	Multiple sclerosis	Enema	Phase 2	Terminated	Canada
NCT03594487	Multiple sclerosis	Colonoscopy	Phase 1	Recruiting	United States
NCT03975413	Multiple sclerosis	Not applicable	Not applicable	Active, not recruiting	United States
NCT04203017	Multiple sclerosis	Capsules	Phase 1	Recruiting	Russian Federation
NCT03691987	Chronic fatigue syndrome/myalgic encephalomyelitis	Enema	Phase 2	Recruiting	Norway
NCT04158427	Chronic fatigue syndrome/myalgic encephalomyelitis	Colonoscopy	Not applicable	Enrolling by invitation	Finland
NCT03233100	Depressive symptoms, anxiety symptoms, gut-brain disorders	Not applicable	Not applicable	Unknown status∗	China
NCT03281044	Major depressive disorder	Capsules	Phase 2	Terminated	Switzerland
NCT04001439	Depression in schizophrenia	Capsules	Not applicable	Not yet recruiting	France
NCT03998423	Alzheimer disease	Capsules	Phase 1	Terminated	United States
NCT02889627	Epilepsy	Microbiota suspension infused into midgut or lower gut (no detail)	Phase 2, phase 3	Recruiting	China
NCT03279224	Bipolar depression	Colonoscopy	Phase 2, phase 3	Recruiting	Canada
NCT03766321	Amyotrophic lateral sclerosis	Nasojejunal	Not applicable	Recruiting	Italy
NCT04132427	Pitt-Hopkins syndrome	Oral (no detail)	Phase 2	Recruiting	United States
NCT03416751	Alcohol abuse	Enema	Phase 1	Completed	United States
NCT03928808	Anorexia nervosa	Nasogastric tube	Early phase 1	Suspended	United States
NCT02336789	Disorientation as to people, time and place	Colonoscopy	Not applicable	Unknown status∗	Israel
NCT04014413	Hepatic encephalopathy, multiple sclerosis, autism, alcohol dependence	Not applicable	Not applicable	Recruiting	China

^∗^Study has passed its completion date, and status has not been verified in more than two years. Date from https://clinicaltrials.gov/.

## Data Availability

Data from the review are available upon request from the corresponding authors (Y.Q.N. and Y.J.Z.).
